# GABA_A_ receptors: structure, function, pharmacology, and related disorders

**DOI:** 10.1186/s43141-021-00224-0

**Published:** 2021-08-21

**Authors:** Amr Ghit, Dina Assal, Ahmed S. Al-Shami, Diaa Eldin E. Hussein

**Affiliations:** 1 Department of Biology and Biotechnology, University of Pavia, Pavia, Italy; 2 Department of Biotechnology, Institute of Graduate Studies and Research (IGSR), Alexandria University, Alexandria, Egypt; 3 Department of Biotechnology, American University in Cairo (AUC), Cairo, Egypt; 4 Department of Zoology, Faculty of Science, Alexandria University, Alexandria, Egypt; 5 Animal Health Research Institute (AHRI), Agricultural Research Center (ARC), Port of Alexandria, Alexandria, Egypt

**Keywords:** GABA, GABA_A_R, Benzodiazepine, Barbiturates, Allosteric modulation, Autism spectrum disorder, Alzheimer’s disease, Epilepsy, Schizophrenia

## Abstract

**Background:**

γ-Aminobutyric acid sub-type A receptors (GABA_A_Rs) are the most prominent inhibitory neurotransmitter receptors in the CNS. They are a family of ligand-gated ion channel with significant physiological and therapeutic implications.

**Main body:**

GABA_A_Rs are heteropentamers formed from a selection of 19 subunits: six α (alpha1-6), three β (beta1-3), three γ (gamma1-3), three ρ (rho1-3), and one each of the δ (delta), ε (epsilon), π (pi), and θ (theta) which result in the production of a considerable number of receptor isoforms. Each isoform exhibits distinct pharmacological and physiological properties. However, the majority of GABA_A_Rs are composed of two α subunits, two β subunits, and one γ subunit arranged as γ2β2α1β2α1 counterclockwise around the center. The mature receptor has a central chloride ion channel gated by GABA neurotransmitter and modulated by a variety of different drugs. Changes in GABA synthesis or release may have a significant effect on normal brain function. Furthermore, The molecular interactions and pharmacological effects caused by drugs are extremely complex. This is due to the structural heterogeneity of the receptors, and the existence of multiple allosteric binding sites as well as a wide range of ligands that can bind to them. Notably, dysfunction of the GABAergic system contributes to the development of several diseases. Therefore, understanding the relationship between GABA_A_ receptor deficits and CNS disorders thus has a significant impact on the discovery of disease pathogenesis and drug development.

**Conclusion:**

To date, few reviews have discussed GABA_A_ receptors in detail. Accordingly, this review aims to summarize the current understanding of the structural, physiological, and pharmacological properties of GABA_A_Rs, as well as shedding light on the most common associated disorders.

## Background

γ-Aminobutyric acid (GABA), the primary inhibitory neurotransmitter in the central nervous system (CNS), is a key coordinator of brain activity. GABA’s inhibitory effects are mediated by two types of receptors, GABA_A_ and GABA_B_ receptors [1]. GABAergic neurotransmission is critical in neurodevelopmental disorders [2]. GABA_A_R is one of the most significant drug targets in the treatment of neuropsychiatric disorders such as epilepsy, insomnia, and anxiety, as well as in anesthesia in surgical operations [3]. In addition, genetic studies have documented the relationship between GABA_A_R subunit genes and epilepsy [4], eating disorder [5], autism [6, 7], and bipolar disorders [8]. GABA_B_ receptors are members of the C family of G protein-coupled receptors (GPCRs), which are found in the nervous system and have been linked to some neurological and psychiatric disorders [9]. They are structurally and functionally distinct from GABA_A_ receptors and will not be covered in this article. GABA_C_ receptors are now considered to be part of GABA_A_ receptor isoforms that are entirely made up of rho (ρ) subunits [10]. In this review, we will try to provide a quick rundown of what we know about GABA_A_ receptors, including their structure, function, pharmacology, and related disorders.

## GABA_A_Rs structure and gene organization

**Fig. 1:**
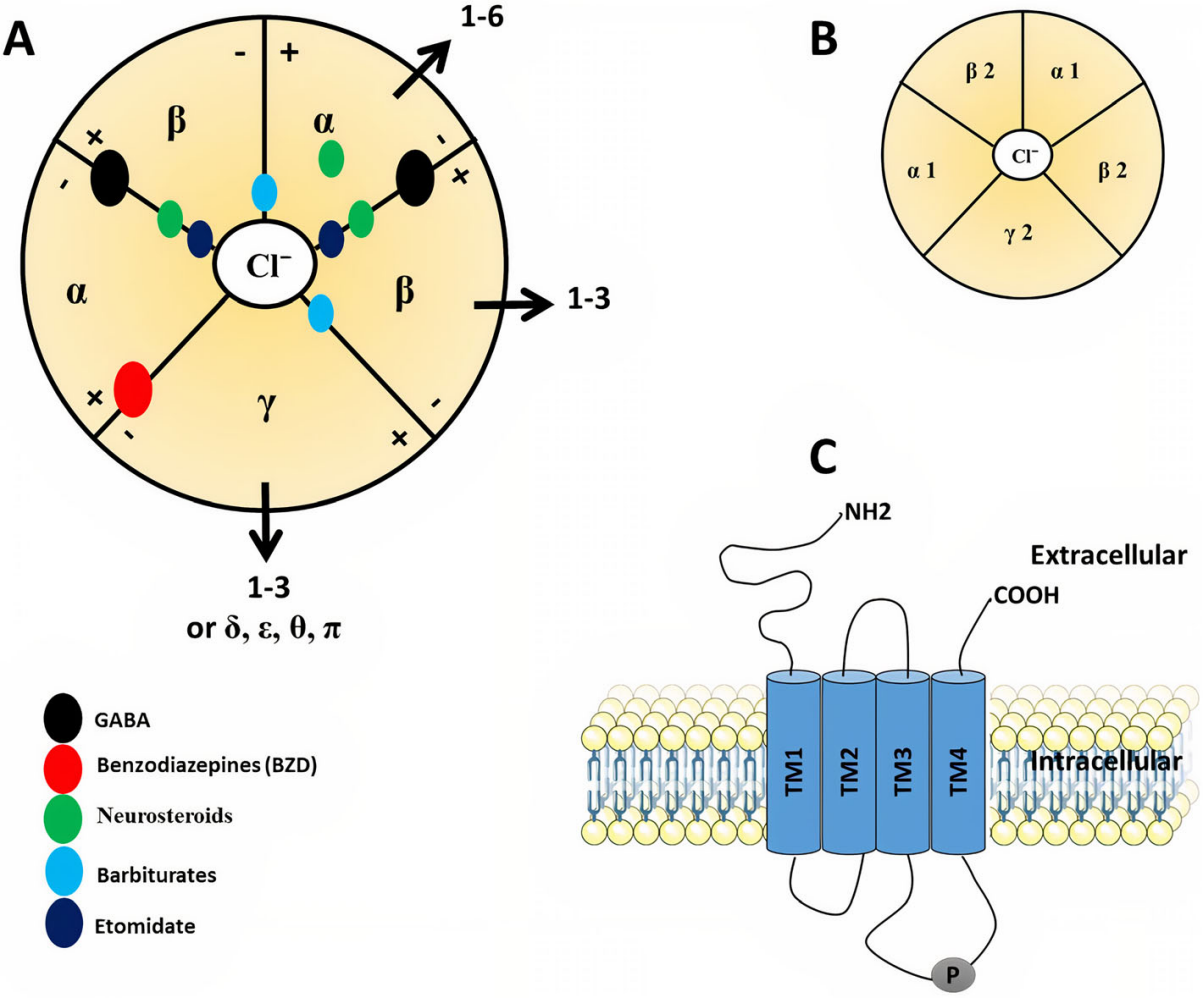
Schematic representation of GABA_A_ receptor structure. (**A**) GABA_A_ receptors are heteropentamers that form a chloride-ion-permeable channel. They are formed by 19 subunits: α1–6, β1–3, γ1–3, δ, ε, θ, π, and ρ1–3. The GABA binding sites are located at the junction of β+/α−, whereas benzodiazepines (BZs) are located at α+/γ− interface. Anesthetics are located at different sites where barbiturates bind to α+/β−, and γ+/β− interfaces while etomidate binds to β+/α− interface. The binding site of the neurosteroids is located at α subunit as well as the β+/α− interface. (**B**) The most popular GABA_A_R isoform is composed of α1, β2, and γ2 subunits arranged γ2β2α1β2α1 counterclockwise around the central pore. (**C**) The mature subunit contains a large hydrophilic extracellular N-terminal, four hydrophobic transmembrane domains (TMD: TM1–TM4), and a small extracellular C terminus. TM1 and TM2 are connected by a short intracellular loop while a short extracellular loop connects TM2 and TM3. Besides, TM3 and TM4 are connected by a lengthy intracellular loop that can be phosphorylated

GABA_A_ receptors are ligand-gated chloride channels that consist of pentameric combinations of different subunits. A total of 19 GABA_A_ receptor subunit genes have been identified in humans that code for six α (alpha1-6), three β (beta1-3), three γ (gamma1-3), three ρ (rho1-3), and one each of the δ (delta), ε (epsilon), π (pi), and θ (theta) (Fig. 1A; Table 1) [11–13]. The diversity of GABA_A_ receptors is due to the alternative splicing of several genes [14]. The GABA_A_ receptor subunit genes are mainly arranged into four clusters on the human genome’s chromosomes 4, 5, 15, and X. Four genes, α2, α4, β1, and γ1 on chromosome 4; four genes α1, α6, β2, and γ2 on chromosome 5; three genes, α5, β3, and γ3 on chromosome 15; and three genes, α3, ϵ, and θ on chromosome X (Table 1) [15]. The receptor composition and arrangement influence its functional and pharmacological properties [16, 17].

**Table 1:** GABA_A_ receptor subunits

Receptor subunit	Gene	Chromosome	Location	Reference
GABA-A alpha 1 (α1)	GABRA1	5	5q34	Gene ID: 2554
GABA-A alpha 2 (α2)	GABRA2	4	4p12	Gene ID: 2555
GABA-A alpha 3 (α3)	GABRA3	X	Xq28	Gene ID: 2556
GABA-A alpha 4 (α4)	GABRA4	4	4p12	Gene ID: 2557
GABA-A alpha 5 (α5)	GABRA5	15	15q12	Gene ID: 2558
GABA-A alpha 6 (α6)	GABRA6	5	5q34	Gene ID: 2559
GABA-A beta 1 (β1)	GABRB1	4	4p12	Gene ID: 2560
GABA-A beta 2 (β2)	GABRB2	5	5q34	Gene ID: 2561
GABA-A beta 3 (β3)	GABRB3	15	15q12	Gene ID: 2562
GABA-A gamma 1 (γ1)	GABRG1	4	4p12	Gene ID: 2565
GABA-A gamma 2 (γ2)	GABRG2	5	5q34	Gene ID: 2566
GABA-A gamma 3 (γ3)	GABRG3	15	15q12	Gene ID: 2567
GABA-A delta (δ)	GABRD	1	1p36.33	Gene ID: 2563
GABA-A epsilon (ε)	GABRE	X	Xq28	Gene ID: 2564
GABA-A pi (π)	GABRP	5	5q35.1	Gene ID: 2568
GABA-A theta (θ)	GABRQ	X	Xq28	Gene ID: 55879
GABA-A rho 1 (ρ1)	GABRR1	6	6q15	Gene ID: 2569
GABA-A rho 2 (ρ2)	GABRR2	6	6q15	Gene ID: 2570
GABA-A rho 3 (ρ3)	GABRR3	3	3q11.2	Gene ID: 200959

Data are compiled from NCBI-Gene

Each subunit has been thoroughly investigated in terms of amino acid sequence, level of expression, and localization in brain tissues, but it is still unclear the interaction between them to form many different isoforms [18]. This variety of isoforms may be present even in a single cell [10]. However, it is widely assumed that the main adult isoform is composed of α1, β2, and γ2 subunits which are arranged γ2β2α1β2α1 counterclockwise around a central pore as viewed from the cell exterior (Fig. 1B) [19].

GABA_A_R subunits share a common structure (Fig. 1A). The mature subunit is composed of ∼450 amino acid residues. It contains N-terminal, a large hydrophilic extracellular domain (ECD), four hydrophobic transmembrane domains (TMD: TM1–TM4) where TM2 is believed to form the pore of the chloride channel, and intracellular domain (ICD) between TM3 and TM4 which is the site of protein interactions and post-translational modifications that modulate receptor activity (Fig. 1C) [20, 21]. The neurotransmitter GABA, as well as psychotropic drugs such as benzodiazepines (BZDs), bind to the N-terminal at binding sites α-β and α-γ interfaces, respectively. Neurosteroids and anesthetics like barbiturates, on the other hand, are found within the TMD of α and β subunits (Fig. 1A) [22–25].

## GABA_A_Rs distribution

In the CNS, some GABA_A_R subunits possess broad expression while other subunits exhibit restricted expression. For example, the α6 subunit is expressed only in the cerebellum while the ρ subunit is expressed mainly, but not exclusively, in the retina [26]. GABA_A_ receptors localized to postsynaptic sites in the brain are mainly composed of the α1–3, β1–3, and γ2 where GABA neurotransmitter can bind with and open chloride channels, thus increasing the anion conductance for a short period (milliseconds), leading to hyperpolarization of a depolarized membrane. This type of GABA inhibition has been termed phasic inhibition. On the other hand, GABA_A_ receptors composed of the α4–6, β2/3 and δ subunits can localize to extrasynaptic sites where the low GABA concentration can open these receptors for a longer period which is called tonic inhibition [27]. The most popular isoforms of extrasynaptic GABA_A_Rs mediating tonic inhibition are α4βδ receptors in the forebrain, α6βδ receptors in the cerebellum and α1βδ receptors in the hippocampus [28]. It has been found that α2, α3, and β3 subunit-containing receptors are ~100 times more concentrated at synapses than in the extrasynaptic membrane [29]. Not all γ2-containing receptors are concentrated postsynaptic for example, α5βγ2 receptors are found at extrasynaptic sites involved in tonic inhibition [28]. Apart from phasic and tonic inhibition, the γ2 subunit is essential for postsynaptic clustering of GABA_A_ receptors [30] and the γ3 subunit substitutes γ2 to contribute to the development of the postnatal brain [31]. On the other hand, outside the CNS, GABA_A_ receptors have been found in different types of immune cells [32, 33], liver cells [34], pancreatic islet β-cells [35], and airway smooth muscle [36]. Despite these observations, the laws that regulate GABA_A_Rs assembly, as well as the exact process by which GABA_A_R isoforms are distributed, remain unknown.

## GABA neurotransmission

**Fig. 2:**
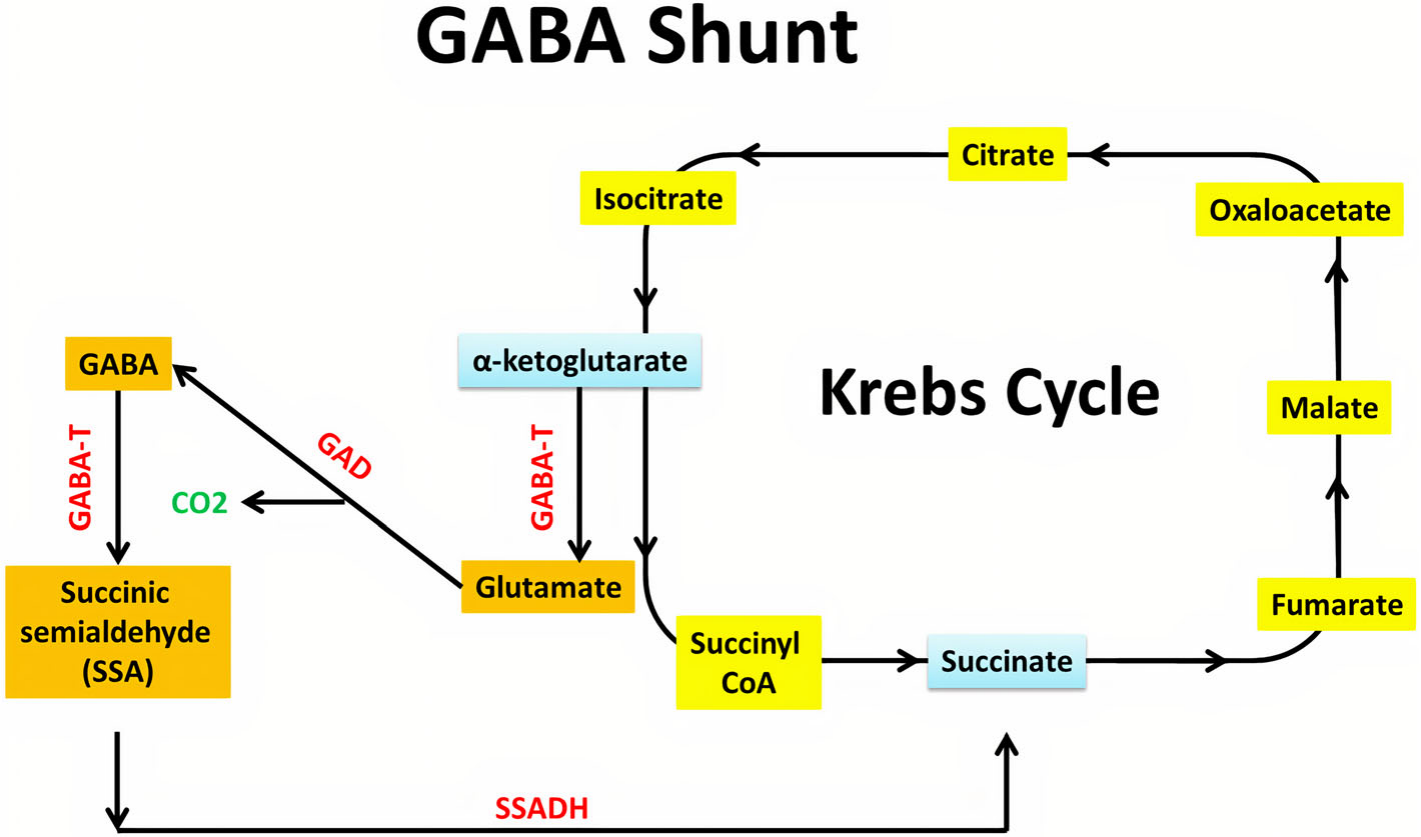
Schematic illustration of GABA shunt. Transamination of α-ketoglutarate by GABA-α ketoglutarate transaminase (GABA-T) to produce glutamate which is decarboxylated to GABA by glutamic acid decarboxylase (GAD). GABA-T metabolizes GABA to succinic semialdehyde which is oxidized to succinate by succinic semialdehyde dehydrogenase (SSADH). Then, succinate can enter the Krebs cycle and complete the loop

In 1950, Eugene Roberts and Sam Frankel discovered the major inhibitory neurotransmitter in the CNS of mammals, GABA [37]. Glucose is the main precursor for GABA synthesis, even though other amino acids and pyruvate act as precursors. The GABA shunt is a closed-loop system that produces and conserves GABA (Fig. 2). In GABA shunt, the first step is transamination of α-ketoglutarate produced from the metabolism of glucose in the Krebs cycle, by GABA-α ketoglutarate transaminase (GABA-T) to produce l-glutamic acid. Glutamic acid is decarboxylated to GABA by glutamic acid decarboxylase (GAD). GAD is an enzyme that uses vitamin B6 (pyridoxine) as a cofactor and is only expressed in cells that use GABA as a neurotransmitter. GABA-T metabolizes GABA to succinic semialdehyde. This transamination happens when α-ketoglutarate is present, it accepts the amino group extracted from GABA, and reforms glutamic acid. Succinic semialdehyde dehydrogenase (SSADH) oxidizes succinic semialdehyde to succinate. It can enter the Krebs cycle, thereby completing the loop [38]. A vesicular transporter helps to package newly synthesized GABA into synaptic vesicles. SNARE complexes help dock the vesicles into the plasma membrane of the cell [39]. Presynaptic neuron depolarisation releases GABA to the synaptic cleft and diffuses toward postsynaptic receptors. It can bind to post-synaptic GABA receptors (GABA_A_ and GABA_B_), which modulate ion channels, hyperpolarize the cell, and prevent action potential transmission. Regardless of binding to GABA_A_ or GABA_B_ receptors, GABA serves as an inhibitor. In the case of GABA_A_ ionotropic receptor, the presence of GABA increases chloride ion conductance into the cell. Consequently, the increased chloride ion influx results in membrane hyperpolarization, and neuronal excitability is reduced [40]. GABA can then be passed into three pathways. The first one is that GABA can be degraded extracellularly by GABA-T into succinate semialdehyde which then enters the citric acid cycle. The second is that the GABA can be reuptaken to nerve terminals for utilization again. The third one is that the GABA can be reuptaken to the glial cell where it undergoes metabolism to succinic semialdehyde by GABA-T or it becomes glutamine which is transported to neurones, where it is converted to glutamate by glutaminase and re-enters GABA shunt. In glia, GABA cannot be synthesized again from glutamate due to the absence of GAD [41, 42].

## The physiological role of GABA and GABA_A_ receptors

Certainly, GABA/GABA_A_Rs signaling is the most prominent inhibitory pathway in the CNS. As we discussed before, there are two forms of GABA inhibition: phasic and tonic inhibition. The transient stimulation of GABA_A_ receptors by GABA reduces postsynaptic neuron excitability, resulting in phasic inhibition [43, 44]. Tonic inhibition, on the other hand, is thought to be a continuous mechanism of inhibition that regulates excitation through long-term hyperpolarization [45]. Tonic inhibition plays an important role in synaptic plasticity, neurogenesis [46, 47] as well as cognitive functions [48, 49]. Any disturbance in phasic or tonic inhibition is associated with many neurological and psychiatric diseases. Thus, modulating these signals has become the basis of drug therapy as well as anesthesia [50–55].

Furthermore, the GABA_A_ receptor plays a pivotal role in neuronal cell proliferation and fate determination. A pioneering study showed that depolarizing GABA actions leads to a decrease in both DNA synthesis and the number of bromodeoxyuridine (BrdU)-labeled cells at the subventricular zone (SVZ) that mean GABA can affect the proliferation of progenitor cells in rat embryonic neocortex [56]. Furthermore, GABA or muscimol, a GABA_A_ receptor agonist, also triggers membrane depolarization and induces proliferation of postnatal cerebellar granule progenitor cells in the developing rat cerebellum [57]. In the adult hippocampus, the neuronal progenitor cells at the subgranular zone (SGZ) show tonic GABAergic conductance. Impairment of this conductivity, as well as the increase in newly generated cells labeled by BrdU, was induced by genetic deletion of GABA_A_Rs containing α4, but not δ subunits [47, 58, 59]. In the postnatal subventricular zone (SVZ), GABA limits the proliferation of glial fibrillary acidic protein (GFAP)-expressing progenitors thought to be stem cells (also called Type 1 cells) [60]. Also, a recent study suggested that GABA_A_ receptor contributes to determining the cell fate of neural stem cells [61]. These results indicate that adult neurogenesis may be influenced by multiple functions of GABA_A_ receptors as well as ambient GABA released in an autocrine/paracrine manner [62, 63].

Of note, GABA_A_ receptors have additional physiological functions in tissues and organs outside the nervous system [64]. Such as in the pancreatic islet, β-cells synthesize huge amounts of GABA [35]. Via GABA_A_ receptors, GABA suppresses glucagon secreted by α-cells [65], and increases insulin secreted by β-cells [66]. In addition, GABA stimulates β-cells proliferation and growth [66, 67]. Therefore, targeting GABA/GABA_A_ signaling is likely to be a part of diabetes treatment [68].

## Molecular pharmacology of GABA_A_ receptors

Apart from GABA, a variety of ligands have been discovered that bind to various locations on the GABA_A_R and regulate it. Binding sites are located at particular receptor subtypes, and these subtypes determine the receptors’ distinct pharmacological fingerprints [69]. The GABA-binding site, also known as the active site or orthosteric site, is where orthosteric agonists and antagonists bind. Orthosteric agonists, such as GABA, gaboxadol, isoguvacine, muscimol, and progabide [70–72], activate the receptor, resulting in increased Cl^−^ conductance. By contrast, orthosteric antagonists, such as bicuculline and gabazine [73], compete with GABA for binding, inhibiting its effect and lowering Cl^−^ conductance. Allosteric modulators, on the other hand, bind elsewhere on the receptor and exert their effect by causing conformational changes in the receptor either positively (PAM) such as barbiturates, benzodiazepines, z-drugs (nonbenzodiazepines) alcohol (ethanol), etomidate, glutethimide, anesthetics, and certain neurosteroids, or negatively (NAM) such as pregnenolone sulfate and zinc [54, 74, 75]. Non-competitive chloride channel blockers (ex., picrotoxin) are ligands that bind to or near the central pore of the GABA_A_R and block Cl^−^ conductance [76]. Moreover, silent allosteric modulators (SAM) are a class of GABA_A_R modulators that can compete with a PAM or a NAM for the occupation of the binding site such as flumazenil [75, 77]. The characteristics of ligands that contribute to receptor activation are usually used as anxiolytic, anticonvulsant, sedative, and muscle relaxant drugs. On the other side, ligands that inhibit receptor function usually have opposite pharmacological effects such as convulsion and anxiogenesis [78, 79]. Interestingly, some subtypes of NAM (ex., α5IA) are being studied for their nootropic properties as well as potential therapies for GABAergic medication adverse effects [80].

### GABA and GABA analogs

Cys-loop receptors typically have their neurotransmitter binding site at the extracellular interface between two neighboring subunits. The binding site’s principal face (+) is made up of three loops (A, B, and C), whereas the complementary face (−) comprises three β-strands and one loop (D, E, F, or G) [81, 82]. In GABA_A_Rs, αβγ subtype (2α:2β:1γ) has two GABA binding sites at the β +/α − interfaces (Fig. 1A). When GABA occupies just one site, the channel opens; however, when both sites are occupied, the chances of channel opening rise dramatically [83]. Besides, chemicals with similar structures to GABA can attach to GABA binding sites and give different effects such as muscimol (agonist), gaboxadol (partial agonist), and bicuculline (competitive antagonist) [82].

Actually, it is still a mystery how amino acid residues interact with GABA. However, in a previous study based on αβγ subtype, GABA formed hydrogen bonds with α1T129 and β2T202, salt bridges with α1R66 and β2E155, and cation–pi interaction with β2Y205 [84]. On the other hand, β +/α− interface has aromatic residues formed by βY97, βY157, βF200, βY205, and αF64 which are conserved at the β +/β −, β +/γ −, and β +/δ − interfaces. Furthermore, the GABA-binding subunit residues R131, T129, and L127 are maintained at the equivalent places in the β, γ, and δ subunits [81, 84, 85]. Future studies will examine whether GABA and other structurally similar chemicals are attracted to these non-canonical sites, as well as how these sites may influence receptor activation.

### Benzodiazepines

Benzodiazepines (BZDs) are commonly used in different treatments related to anxiety, sleep disorders, seizure disorders, muscle spasms, and some forms of depression [86]. BZD allosterically modulate GABA_A_R and give its therapeutic effect through binding to the α+/γ − interface (Fig. 1A) and increasing Cl^−^ conductance [24, 87]. Interestingly, amino acids involved in the binding sites of BZDs are homologous to that of the GABA binding site at the β +/α − interface [88]. Besides, mutations that converted histidine to arginine (α1H101R, α2H101R, α3H126R, and α5H105R) at the β2γ2 subtype of GABA_A_Rs eliminated diazepam activity, while reverse mutations (from R to H) elicited the diazepam response [89]. BZD-sensitive GABA_A_Rs subtypes are formed of two α subunits with two β subunits and a γ subunit (Fig. 1A) [90]. Likewise, GABA_A_R containing α4, α6, and γ2 subunits, potently bind many BZD ligands [91, 92]. But subtypes containing δ are relative with low abundance, and the subunits replacing γ and δ, such as ε, are even rarer [93]. Of note, the GABA_A_R subtypes containing δ subunits are located extrasynapically inducing tonic inhibitory currents in major cell populations including cerebellar and hippocampal granule cells [43, 93]. It was thought that these subtypes are not capable to bind any BZD ligands, lacking the high-affinity α+/γ− (site 1), but later it was found to bind some BZD ligands with lower affinity at distinct other sites on the GABA_A_R [54].

Benzodiazepines as zolpidem (an imidazopyridine) and other clinically used hypnotics like zaleplon (a pyrazolopyrimidine) and zopiclone (a cyclopyrrolone), as well as quinolones, triazolopyridazines, and beta-carbolines show a higher affinity for α1-containing receptors than for α2- or α3-containing subtypes, while they do not affect α5-containing GABA_A_Rs [93, 94]. Also, imidazobenzodiazepine oxazole derivatives have shown some α2/α3 selectivity [95]. Pyrazoloquinolinones, which are examples for BZD site-active PAM in γ–containing subtypes, demonstrate a wide range of effects as well as selectivity for α and β subunits [54]. Also, BZD-site ligands have more or less efficacy than traditional BZD agonists on the traditional BZD-sensitive subtypes, and unexpected efficacy on the diazepam-insensitive subtypes like GABA_A_R containing α4 or α6, or α and β without γ [96, 97].

Alpha5IA is selective inverse agonists that bind to the BZD site at the α5 subtype that is highly expressed in the CA1 region of the hippocampus. It has been suggested to improve cognitive functions [98]. Such α5 inverse agonists also reduce side effects of BZDs, general anesthetics [99], and alcohol [100]. They may be useful for treating Down syndrome, autism spectrum disorder, schizophrenia, and affective disorders [101].

### Anesthetics

GABA_A_Rs are remarkable targets of variable volatile anesthetics, intravenous anesthetics, etomidate, and propofol, as well as steroid anesthetics, barbiturates, and ethanol [102]. Anesthetic binding sites on the GABA_A_R can be identified using site-directed mutagenesis [103], substituted cysteine modification protection (SCAMP) [104], or photo-affinity labeling [102, 105]. At higher concentrations, some anesthetics, especially the intravenous anesthetics, etomidate, propofol, and barbiturates, could directly activate GABA_A_Rs in the absence of GABA. Such direct activation distinguished them as GABA-mimetic from benzodiazepines which lack this property. Studies that were based on site-directed mutagenesis produced several residues of interest, particularly in the trans-membrane regions of the α and β subunits, for both volatile and intravenous anesthetics [106].

Of note, methionine residues, especially αM236 and βM286 located in the M1 and M3 domains respectively, have been shown to be significant determinants of etomidate binding and function in experiments that used mutagenesis and photoreactive etomidate analogs. Based on crystal structures of GABA_A_Rs, αM236 and βM286 are expected to be found at the β +/α − interfaces in the TMD, below the GABA binding sites (Fig. 1A). Also, αT237 (M1), αI239 (M1), αL232 (M1), βV290 (M3), and βF289 (M3) are among the additional residues linked to etomidate binding and function [107, 108]. Besides, in α1β3γ2 GABA_A_Rs, other anesthetic binding sites including α +/β − and γ +/β – interfaces (Fig. 1A) have been identified using photoreactive analogs of barbiturate where αA291 (M3), αY294 (M3), βM227 (M1), and γS301 (M3) were among the binding residues [82, 109]. Moreover, in the TMD of β3 homomeric GABA_A_Rs at β +/β – interface, photoreactive propofol can bind to β (+) M286, β (+) F289, and β (–) M227 residues inducing functional activity of the receptor [110–112].

It has been found that β2 and β3 subunits were significant for modulation of GABA_A_R by i.v. anesthetics. In addition, transgenic mice that were generated through β_2_ (N265S) and β_3_ (N265M) mutations in the GABA_A_R became insensitive to the actions of propofol and etomidate [113, 114]. The affinity and efficacy of barbiturate depend on the composition of the subunit, but the α subunit seems to be more important than β [115]. Recently, it has been suggested that the binding of barbiturate, etomidate, and propofol is predominantly at the αβ+/α−γ interface as well as the α+/β− or α+/γ− TMD interfaces in α1β2γ2 [69, 116]. Other photo-affinity labeling depending studies suggested that binding sites for barbiturates and etomidate at α4β3δ GABA_A_R subtypes at the β+/α–, and β+/β– TMD interfaces, respectively, were not suitable for binding of delta selective compound 2 (DS2) or alphaxalone [117].

### Neurosteroid

Endogenous steroids exhibit GABA_A_R-mediated neuroactive effects including anesthesia, anticonvulsant, analgesia, and sedation. The most common examples are allopregnanolone and its synthetic analogs [118]. Although the exact position of the neurosteroid binding sites has yet to be determined, many residues in the TMDs have been shown to impact neurosteroid activity, such as αS240 (M1), αQ241 (M1), αN407 (M4), αY410 (M4), αT236 (M1), and βY284 (M3) [119–121]. The modulatory and activation sites are located at the TMDs of α subunit and β +/α – interfaces respectively (Fig. 1A) [82, 122].

### Flavonoids

Flavonoids are present in most plants and a few microorganisms. They have been discovered as modulators of the BZD-site of GABA_A_Rs, but the variability of compounds within this group participated in showing their potential action at more than one additional binding site on GABA_A_Rs. Flavonoids can act as either negative, positive, or neutralizing on GABA_A_Rs or directly as allosteric agonists [123]. Flavonoids share the elementary structure of a phenylbenzopyran, most commonly of a flavan (2-phenylchromane). Subgroups contain isoflavones, flavonoles, flavones, flavanonole, flavanones, and flavanoles. Among these groups, isoflavones and flavones particularly have been found to interact with the binding site of BZD [124]. Structure-activity experiments have illustrated that flavones have higher potency on BZD radioligand binding than their flavanone or flavonol counterparts. Besides, glycosylation had a negative influence on binding [125]. Flavonoids can also interact with flumazenil-sensitive or -insensitive GABA_A_Rs [123]. Some of the flavonoids have shown subtype-selectivity like flavan-3-ol ester Fa131 [126] or 6,2′- dihydroxyflavone [127]. The flavone hispidulin showed potent activity in crossing the blood-brain barrier associated with the α6β2γ2 subtype of GABA_A_Rs, which is used to reduce the susceptibility of seizures [128].

### Cannabinoids

Cannabinoids are chemical substances present in the cannabis plant. The phytocannabinoid tetrahydrocannabinol (THC) is the primary psychoactive compound in cannabis. Besides, cannabidiol (CBD) is another significant component of the plant [129]. It has been found that CBD has sedative, anxiolytic, and anticonvulsant effects and has been suggested for treating pediatric epilepsies such as Dravet syndrome [130]. CBD, also, showed a low affinity for the main cannabinoid receptor and exhibits an activity profile similar to that of GABA PAMs inducing anxiolytic and anticonvulsant effects [131].

Endocannabinoids, such as 2-Arachidonoylglycerol (2-AG), 2-Arachidonyl glyceryl ether, N-Arachidonoyl dopamine (NADA), Arachidonoylethanolamine (AEA), and Lysophosphatidylinositol (LPI) [132], are substances produced in the body activating cannabinoid receptors (CB1, CB2) [133, 134]. Additionally, they have been identified as positive modulators for GABA_A_R subtypes [135]. Studies on recombinant receptors showed that 2-AG increases GABA_A_R activity at low non-saturating GABA concentrations while decreasing the activity at high saturating GABA concentrations. Therefore, the impact of endocannabinoids on GABA_A_R depends on the regulation of GABA inhibition [136].

### Picrotoxin

Picrotoxin is a plant-derived product, with a universal efficacy as GABA_A_R’s chloride channel blocker. Picrotoxin is found naturally in the *Anamirta Cocculus* plant, although it can be synthesized chemically [137, 138]. It has been utilized as a CNS stimulant, and antidote for poisoning by CNS depressants and barbiturates [139]. However, due to the toxicity of picrotoxin, it is currently used only in research. Furthermore, numerous studies indicated that a wide range of molecules from various chemical families had an affinity for picrotoxin-binding sites such as *t-*butylbicyclophosphorothionate (TBPS), *t*-butylbicycloorthobenzoate (TBOB), pentylenetetrazole, and some insecticides (ex., dieldrin and lindane) [140–142]. A study by Othman et al. (2012) [143] found that low concentrations of GABA increase picrotoxin and TBPS binding affinity to GABA_A_R containing α1β2γ2, while application of GABA at high concentration reduces their binding affinity to the receptor reducing channel blocking activity. This indicates that picrotoxin and ligands of picrotoxin-binding sites are highly dependent on the regulation of GABA inhibition.

### Pharmacology of δ-containing GABA_A_Rs

The unique role of the δ subunit in extra-synaptic GABA_A_Rs, a group of receptors responsible for tonic GABAergic inhibition has generated immense therapeutic and research interests. However, the complicated properties of the δ subunit assembly and the rarity of δ-selective ligands are the main reasons hindering progress in pharmacological studies of these receptors. Variable compounds have been claimed to be selective for the δ subunit. The hypnotic drug THIP (4,5,6,7-tetrahydroisoxazolo[5,4-c]pyridin-3-ol) and gaboxadol are examples of compounds that are known by their direct activation of αβδ with higher efficacy and potency than αβγ but does not discriminate between αβ and αβδ receptors [144, 145]. Similar to THIP, anesthetics, as well as neurosteroids, also show more pronounced action at δ-containing GABA_A_Rs, but their activity is independent of subunit composition, these compounds are not considered to be δ-selective. In contrast, 4-chloro- N-(2-thiophen-2-ylimidazo[1,2-a] pyridin-3-yl) benzamide which was found to be a positive modulator at α4/6βδ, has limited efficacy at αβγ and is inactive at αβ GABA_A_Rs [146].

## GABA_A_ receptor dysfunction and neuro-psychiatric disorders

### Epilepsy

Epilepsy is a neurological disease characterized by frequent and unexpected seizures caused by abnormal brain electricity, which results in loss of consciousness and unusual behaviors [147]. Around 65 million people are affected worldwide, of all ages and genders [148]. An imbalance between excitation and inhibition induced by impaired GABAergic signaling can trigger various forms of epilepsy [149, 150]. Several studies have demonstrated the importance of GABA_A_ receptors as targets for antiepileptic drugs [45, 151, 152]. Mutations in GABA_A_ receptor subunit genes have been linked to several types of idiopathic epilepsy in which the pathophysiological consequences of the mutations are impairments in the gating characteristics of the channel or receptor trafficking [4]. The severity of the disorder appears to depend on the type of mutation (nonsense, missense, or frameshift), its location in the gene (promoter or protein-coding region), the affected region of the encoded protein (intra-/extracellular or transmembrane) and the affected subunit gene [4]. Some mutations in genes encoding the α1, α6, β2, β3, γ2, or δ subunits of GABA_A_Rs have been detected in both animal models of epilepsy and patients with epilepsy [153, 154]. Likewise, Dravet syndrome, also known as severe myoclonic epilepsy in infancy (SMEI), is a form of epilepsy that affects children at the age of approximately 1 year as a result of mutations in genes encoding the α1, β1, β2, and γ2 subunits of GABA_A_Rs [4, 155]. Of note, several GABA_A_R mutations associated with epilepsy lead to abnormal trafficking of the receptors and thus partially or completely impair their expression on the synaptic plasma membrane [155, 156]. Likewise, a study by Dejanovic et al. [157] discovered a missense mutation in GPHN gene, the gene encoding the gephyrin protein, in a patient with Dravet syndrome. Gephyrin is the main protein that clusters and stabilizes GABA_A_Rs at the inhibitory postsynaptic membranes of the central nervous system [158]. Moreover, during the epileptogenic period, expression of the gephyrin protein decreases gradually in the neocortex before returning to baseline during the chronic phase [159]. These findings suggest that the downregulation of GABA_A_R subunits or their interactors that play a functional role in receptor activity, such as gephyrin, maybe the origin of the disease and thus could be used as drug targets.

### Alzheimer’s disease

Alzheimer’s disease (AD) is one of the primary diseases that cause neurodegeneration. Clinically, AD is marked by significant cognitive deficits and regarded as the most common cause of dementia. The aggregation of misfolded amyloid-beta (Aβ) protein, which forms amyloid plaques in the gray matter of the brain, is the origin of AD pathophysiology. Amyloid plaques, neuronal dysfunction, and tangles of neural fibers are major pathological features of the disease [160, 161]. Several experiments, in both AD patients and mice, have shown that accumulation of misfolded Aβ interferes with GABAergic interneuron activity, causing impaired synaptic communication and loss of neural network activity, which eventually leads to cognitive dysfunction [162–165]. A recent study showed transcriptional downregulation of α1, α2, α3, α5, β1, β2, β3, δ, γ2, γ3, and θ subunits of GABA_A_ receptors, and GAD enzyme in the middle temporal gyrus (MTG) of post-mortem brain samples from AD patients. These alterations impair the balance between excitatory and inhibitory pathways that may lead to cognitive dysfunction in AD [166]. Likewise, in biochemical studies, GABA neurotransmitter levels were substantially lower in the CSF as well as the temporal cortex of Alzheimer’s patients, implying impaired synaptic activity and neuronal transmission [44, 167–169]. Also, a study by Limon et al. [170] showed that most aspects of the GABA system were impaired in the brains of AD patients, such as GABAergic neural circuit, GABA levels, and expression levels of GABA_A_ receptors. Furthermore, in AD mice, activating GABA_A_ receptors with baicalein (positive allosteric modulator of the benzodiazepine site of the GABA_A_R) for 8 weeks significantly reduced Aβ production, improved cognitive function, and decreased pathological features [171]. As a result, GABA_A_ receptors seem to be a potential therapeutic target in the treatment of AD.

### Cervical dystonia

Cervical dystonia (CD) is the most frequent type of adult-onset focal dystonia. It is a neurological disorder marked by involuntary and prolonged muscle contractions that cause irregular postures and neck tremors [172–174]. Studying the pathophysiology of isolated cervical dystonia using different methods such as magnetic resonance spectroscopy (MRS), positron emission tomography (PET), and functional magnetic resonance imaging (f-MRI) demonstrated an alteration in the GABA-mediated inhibitory signaling pathway in the cortical, cerebellar, and basal ganglia regions of the brain [175]. Similarly, a significant number of functional defects have been identified in the thalamus of patients with CD [176], and blocking GABA_A_ receptors in the thalamus triggered CD-like symptoms in monkeys [177]. According to a recent study, GABA levels in the right thalamus were decreased in a sample of adult-onset CD patients, and the availability of GABA_A_ receptors was negatively correlated with disease duration and the severity of dystonia [178].

### Brain injury

Several studies investigated whether GABA signaling pathways are involved in several forms of brain injuries using different stroke mice models. As reported in earlier studies, increasing GABA inhibition has shown a neuroprotective role at stroke onset. In contrast, increased GABAergic tonic inhibition at extrasynaptic GABA_A_ receptors would adversely affect and exacerbate stroke pathology. Also, these findings were in line with study results obtained from knockout mice models lacking either α5-GABA_A_ or δ-GABA_A_ receptors, which have revealed better recovery from stroke than healthy mice models because of GABAergic signaling remission [179, 180].

### Autism spectrum disorder

Autism spectrum disorder (ASD) has three characteristic behavioral features: impaired communication and social deficits, and repetitive behaviors. Several studies concluded an imbalance in the glutamatergic/GABAergic signaling pathways and neuroinflammation process were associated with ASD pathophysiology and were also detected in several ASD mice models [181]. Earlier studies reported the presence of molecular-level cortical abnormalities related to GABAergic signaling dysfunction in the brains of ASD. The excitatory and inhibitory signaling imbalance caused by variations in GABA levels represents one of the characteristic features behind behavioral deficits in autism [182]. Mendez et al. [183] conducted a PET imaging study using a radioactive ligand [11C]-Ro15–4513 VT for tracing levels of GABA_A_ receptor α5 subunits in ASD. The results showed a reduction in GABA_A_ receptors in the brain’s two limbic areas (amygdala and nucleus accumbens) of autism patients. Contrary to previous findings, a recent study demonstrated that the impairment in the GABAergic system in ASD mouse models and autistic patients was not associated with alterations in GABA receptor numbers between healthy and ASD controls, as concluded by an earlier study [184]. Also, a recent meta-analysis was conducted to verify earlier findings supporting the association between different genetic variants of GABA_A_ receptor subunits and the risk of developing autism in children. In conclusion, the study showed no association between GABA receptor subunits (β3, α5, and α3) and child autism [185].

### Schizophrenia

Schizophrenia is a multifactorial major psychiatric disorder whose etiology has been associated with hundreds of protein-coding genes reported by different genome-wide association studies. Changes in post-translational modifications of various proteins including GABA_A_ receptors and their contribution to schizophrenia pathophysiology were reported [186]. A previous study showed glycosylation changes in multiple protein receptor subunits in the brains of schizophrenic patients, such as AMPA and GABA_A_ receptor subunits [187].

Specifically, several post-mortem brain studies conducted using lectin affinity analysis and enzyme de-glycosylation of GABA_A_ receptors of superior temporal gyrus of schizophrenic brains demonstrated a decrease in high-mannose N-glycans residues of GABA-associated proteins in individuals with schizophrenia that were specific to different GABA_A_ receptor subunits on the ɑ1, ɑ4, β1, β2, and β3 subunits; increased high- mannose N-glycans on β1 subunit; decreased high-mannose N-glycans on ɑ1 subunit; altered total N-glycans on β2 subunits. These N-glycosylation alterations were further associated with abnormal trafficking and localization of β1/ β2 subunits leading to an aberrant inhibitory signaling system observed in schizophrenia [188, 189].

Furthermore, Marques and his co-workers [190] investigated the availability of α5-GABA_A_ receptors in the hippocampus using PET imaging for hippocampal regions schizophrenic and healthy controls. The study results demonstrated a reduction of [^11^C]-Ro15–4513 VT ([^11^C]-Ro15–4513), which is a radioactive tracer used by PET scans to assess the total volume of distribution for α5-GABA_A_ receptors in the hippocampus of untreated schizophrenic patients versus healthy controls. In contrast, there were no differences between healthy control and the second cohort of patients treated with antipsychotics. These findings were also positively correlated with scaling using PANSS (Positive and Negative Syndrome Scale) scores (i.e., is a medical scale system that measures the severity of schizophrenic symptoms).

### Depression

Major depression is one of the debilitating diseases that leads to neurons’ anatomical and functional changes in the brain’s prefrontal cortex and is induced by chronic stress. Earlier studies had concluded that dysfunction in monoaminergic signaling was the main contribution to depression pathophysiology. Lately, accumulating evidence has suggested the potential role of GABAergic signaling dysfunction in predispositions of depression as it has been reported that both depression and chronic stress are associated with an imbalance in inhibition, and excitation of neuronal signaling resulted from a deficiency in neuronal transmission onto the brain’s prefrontal cortex (PFC). This imbalance resulted from the deficient transmission of GABAergic inhibitory signals onto the brain’s excitatory glutamate interneurons. In this context, several studies were conducted to demonstrate the correlation between GABAergic dysfunction and depression. For instance, a study showed using magnetic resonance imaging established decreased GABA and GAD67 levels and alterations in distinct types of GABA receptor subunits in the brains of depressed patients and stressed mice models. Studies conducted on genetically modified depressed mice models lacking specific GABA receptors showed depressive mice behaviors [191].

Data from magnetic resonance imaging MRI studies reported a reduction in hippocampal volume of the brain of depressed patients, which leads to alterations in neural circuits of different areas of the brain related to emotionality, such as amygdala and prefrontal cortex. Interestingly, study results using depressed mice models lacking GABA_A_ receptors showed that any alterations in the brain’s GABAergic system were presented by cognitive, neuroanatomical, and behavioral deficits like significant depression disorder symptoms presented by depressed animal models. Accordingly, it is now presumed that the GABAergic system plays a vital role in controlling neuronal transmission in neuronal maturation in the hippocampus. Therefore, it is considered a therapeutic target for potential antidepressant drugs [28, 192].

### Attention and social behavior

Several studies have shown that inhibiting cortical GABA_A_ receptors causes impaired attention [16, 193–197], social behavior [198], and decision-making [199]. Recently, it has been demonstrated that mice models having impaired 5-alpha GABA_A_ receptors were presented with behavioral deficits like symptoms associated with attention and social disorders [194].

## Conclusion

Deep insights into the different GABA_A_ receptor isoforms’ composition, arrangement, subunit interactors, and molecular pharmacology will give us a clear vision to understand alterations that may lead to CNS disorders. In our view, these discussions are of vital importance in drug discovery and development in the future.

## Data Availability

All data generated or analyzed during this study are included in this manuscript.

## Abbreviations

2-AG: 2-Arachidonoylglycerol
AD: Alzheimer’s disease
AEA: Arachidonoylethanolamine
ASD: Autism spectrum disorder
Aβ: Amyloid beta
BrdU: Bromodeoxyuridine
BZ/BZD: Benzodiazepine
CB: Cannabinoid receptors
CBD: Cannabidiol
CD: Cervical dystonia
DS2: Delta selective compound 2
f-MRI: Functional magnetic resonance imaging
GABA: γ-Aminobutyric acid
GABAARs: γ-Aminobutyric acid sub-type A receptors
GABA-T: GABA transaminase
GAD: Glutamic acid decarboxylase
GFAP: Glial fibrillary acidic protein
GPCRs: G protein-coupled receptors
ICD: Intracellular domain
LPI: Lysophosphatidylinositol
MRS: Magnetic resonance spectroscopy
MTG: Middle temporal gyrus
NADA: N-Arachidonoyl dopamine
NAM: Negative allosteric modulation
PAM: Positive allosteric modulation
PANSS: Positive and Negative Syndrome Scale
PET: Positron emission tomography
PFC: Prefrontal cortex
SAM: Silent allosteric modulators
SCAMP: Cysteine modification protection
SMEI: Severe myoclonic epilepsy in infancy
SSADH: Succinic semialdehyde dehydrogenase
SVZ: Subventricular zone
THC: Tetrahydrocannabinol
TMD: Transmembrane domains
